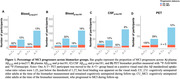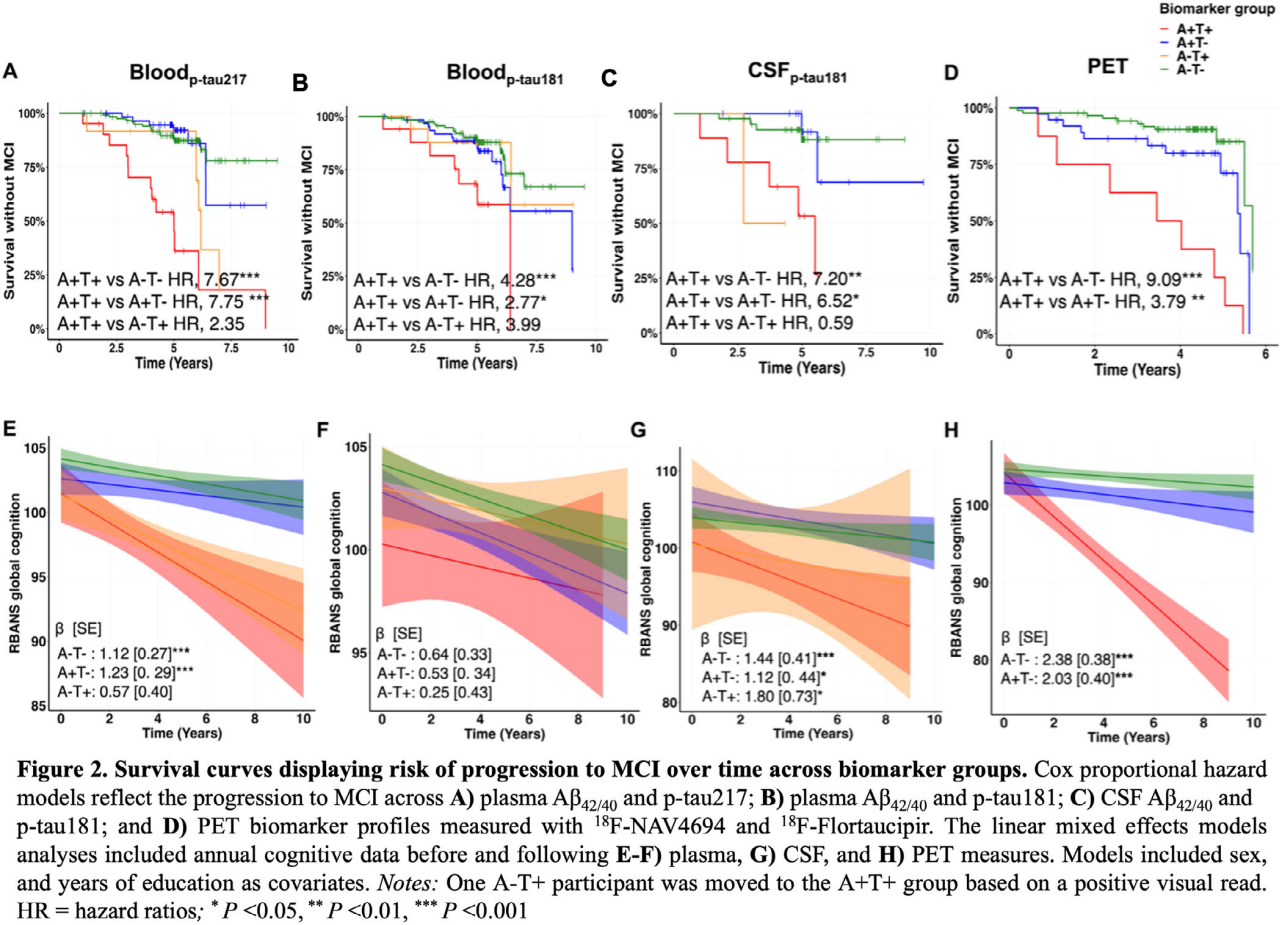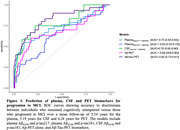# The role of biofluid markers in predicting near‐term cognitive impairment

**DOI:** 10.1002/alz.092241

**Published:** 2025-01-09

**Authors:** Yara Yakoub, Fernando Gonzalez‐Ortiz, Nicholas J. Ashton, Thomas K Karikari, Cherie Strikwerda‐Brown, Frédéric St‐Onge, Valentin Ourry, Michael Schöll, Maiya R. Geddes, Simon Ducharme, Pedro Rosa‐Neto, Jean‐Paul Soucy, John C.S. Breitner, Henrik Zetterberg, Kaj Blennow, Judes Poirier, Sylvia Villeneuve, PREVENT‐AD Research Group

**Affiliations:** ^1^ Douglas Mental Health University Institute, Centre for Studies on the Prevention of Alzheimer's Disease (StoP‐AD), Montréal, QC Canada; ^2^ Institute of Neuroscience and Physiology, University of Gothenburg, Mölndal Sweden; ^3^ Centre for Age‐Related Medicine, Stavanger University Hospital, Stavanger Norway; ^4^ Department of Psychiatry and Neurochemistry, Institute of Neuroscience and Physiology, The Sahlgrenska Academy, University of Gothenburg, Mölndal, Gothenburg Sweden; ^5^ King's College London, London UK; ^6^ NIHR Biomedical Research Centre for Mental Health and Biomedical Research Unit for Dementia at South London and Maudsley, NHS Foundation, London UK; ^7^ Department of Psychiatry, School of Medicine, University of Pittsburgh, Pittsburgh, PA USA; ^8^ School of Psychological Sciences, The University of Western Australia, Perth Australia; ^9^ Department of Psychiatry and Neurochemistry, Institute of Neuroscience and Physiology, The Sahlgrenska Academy, University of Gothenburg, Mölndal Sweden; ^10^ Montreal Neurological Institute, McGill University, Montréal, QC Canada; ^11^ Massachusetts Institute of Technology, Cambridge, MA USA; ^12^ McGill Center for Research Studies in Aging, Montréal, QC Canada; ^13^ Montreal Neurological Institute, Montréal, QC Canada; ^14^ Department of Psychiatry, McGill University, Montréal, QC Canada; ^15^ UK Dementia Research Institute, University College London, London UK; ^16^ UW Department of Medicine, School of Medicine and Public Health, Madison, WI USA

## Abstract

**Background:**

PET biomarkers have proven valuable for identifying cognitively unimpaired (CU) individuals at‐risk of near‐term clinical progression. Given the increasing interest in plasma biomarkers to detect Alzheimer’s pathology, we assessed levels of amyloid (Aβ_42/40_) and tau (p‐tau217 and p‐tau181) biomarkers in plasma (A+T+_plasma_) in CU individuals as predictors of clinical progression to mild cognitive impairment (MCI). We then repeated these analyses using cerebrospinal fluid (CSF) and PET biomarkers.

**Method:**

We studied 218 participants from the PREVENT‐AD cohort with cognitive follow‐up (mean 5.34 years, range 1–10 years) and plasma biomarkers available while CU. Cognition was assessed annually using the RBANS. Plasma biomarkers were measured using IP‐MS for Aβ_42/40_ and in‐house single molecular arrays for p‐tau217 and p‐tau181. An overlapping 71 participants had Aβ_42/40_ (Mesoscale Discovery assay) and p‐tau181 (Innotest immunoassay) CSF data available, and 135 participants had Aβ (^18^F‐NAV4694) and tau (^18^F‐florbetapir) PET data. We also performed receiver operating characteristic (ROC) curves to compare the performance of plasma (Aβ_42/40_ and p‐tau217; Aβ_42/40_ and p‐tau181), CSF (Aβ_42/40_ and p‐tau181) or PET (Aβ‐ and tau‐PET) biomarkers in predicting progression to MCI. We examined an additional model including only Aβ‐PET, given the importance of this technique as a stand‐alone marker for recruitment in anti‐Aβ trials.

**Result:**

The proportion who progressed from CU to MCI was 62% in individuals with A+T+plasma_217_; and 41% with A+T+plasma_181_; 56% with A+T+CSF_181_ and 100% with A+T+PET (Figure 1). Cox proportional hazard models indicated a faster progression rate in all A+T+ groups compared with their matched A‐T‐ biomarker groups (Figure 2). Considering longitudinal RBANS scores, all A+T+ groups declined faster than the other groups, except for the plasma_181_ group. Finally, the PET (Aβ and tau‐PET) and CSF models were superior to the plasma or Aβ‐PET alone in identifying progressors, with no meaningful difference between the Aβ‐PET and the biofluid models (Figure 3).

**Conclusion:**

CU individuals with A+T+ based on plasma biomarkers are at increased risk of cognitive decline and clinical progression. Nevertheless, the proportion who progressed to MCI in individuals classified with plasma was lower than that found using PET. Thus, the latter should remain the gold standard to identify presymptomatic pathological changes.